# Adaptive Lower Limb Pattern Recognition for Multi-Day Control

**DOI:** 10.3390/s22176351

**Published:** 2022-08-24

**Authors:** Robert V. Schulte, Erik C. Prinsen, Jaap H. Buurke, Mannes Poel

**Affiliations:** 1 Roessingh Research & Development, Roessinghsbleekweg 33b, 7522 AH Enschede, The Netherlands; 2 Department of Biomedical Signals & Systems, University of Twente, Drienerlolaan 5, 7522 NB Enschede, The Netherlands; 3 Department of Biomechanical Engineering, University of Twente, Drienerlolaan 5, 7522 NB Enschede, The Netherlands; 4 Department of Data Management & Biometrics, University of Twente, Drienerlolaan 5, 7522 NB Enschede, The Netherlands

**Keywords:** electromyography, machine learning, lower limb, pattern recognition, multi-day

## Abstract

Pattern recognition in EMG-based control systems suffer from increase in error rate over time, which could lead to unwanted behavior. This so-called concept drift in myoelectric control systems could be caused by fatigue, sensor replacement and varying skin conditions. To circumvent concept drift, adaptation strategies could be used to retrain a pattern recognition system, which could lead to comparable error rates over multiple days. In this study, we investigated the error rate development over one week and compared three adaptation strategies to reduce the error rate increase. The three adaptation strategies were based on entropy, on backward prediction and a combination of backward prediction and entropy. Ten able-bodied subjects were measured on four measurement days while performing gait-related activities. During the measurement electromyography and kinematics were recorded. The three adaptation strategies were implemented and compared against the baseline error rate and against adaptation using the ground truth labels. It can be concluded that without adaptation the baseline error rate increases significantly from day 1 to 2, but plateaus on day 2, 3 and 7. Of the three tested adaptation strategies, entropy based adaptation showed the smallest increase in error rate over time. It can be concluded that entropy based adaptation is simple to implement and can be considered a feasible adaptation strategy for lower limb pattern recognition.

## 1. Introduction

Pattern recognition systems based on electromyography (EMG) suffer from issues with robustness and stability of classification accuracy over longer periods of time. The accuracy of myoelectric pattern recognition could change up to 20–30% throughout the day or between days due to the nature of EMG [1,2]. This effect, the reduction of accuracy over time due to a changing input signal, is called concept drift [3]. Concept drift in myoelectric pattern recognition could be caused by fatigue, sensor replacement and varying skin conditions [3] and user adaptability [4]. Therefore, long-time use often requires frequent retraining of myoelectric pattern recognition systems and this forms a barrier for commercial use, for instance in prosthetics [5,6]. He et al. [7,8] investigated long-term use of a myoelectric pattern recognition system over 12 days. The authors showed that the accuracy decreases up to 10% per day and tends to plateau after eight days, but below the required minimum accuracy needed to control a prosthesis. Kaufmann et al. [4] monitored classification accuracy in one subject over 21 days. They observed a gradual decrease in accuracy over time, up to a decrease of almost 40% over 21 days for certain classifiers, possibly due to changes in skin conditions, electrode placement and user adaptability. Therefore, a strategy needs to be implemented to counter the effect of concept drift to use a myoelectric pattern recognition system reliably over multiple days.

Several strategies have been developed to overcome the problem of concept drift in pattern recognition systems. One strategy is to use incremental learning, in which the predicted labels are used to extend the training set [9,10,11]. A downside of this approach is that by using predicted labels, also faulty labels are used to extend the training set [11]. This leads to error accumulation, resulting in poor performance. Next to that, by adding more data to the training set to retrain the algorithm the number of samples would increase, which increases the computational load. If this grows too large, the computational time could be too long, which could harm the overall use of a pattern recognition system due to delays in prediction. Therefore, using a sample selection procedure could be beneficial. By adding only samples which are suitable for adaptation the error would not accumulate and the number of samples in the training set would be limited. Several sample selection procedures have been investigated in literature, such as only keeping the last number of samples [4], a resampling procedure based on clustering [11,12,13], sample selection based on entropy [14,15,16] or sample selection based on backward prediction [17,18,19,20,21,22].

Although many of these adaptation strategies have been applied in the upper limb, the amount of studies into lower limb pattern recognition adaptation are limited. Studies have shown to reach high accuracy in lower limb pattern recognition [23,24,25,26], however, only a few studies have looked into multi-day pattern recognition [27]. Simon et al. [28] investigated the influence of amount of training data in two transfemoral amputees spanning four measurement sessions. They concluded that the error rate in session 4 decreased from 1.45% (0.3 Standard Error of the Mean (SEM)) when using only training data from session 1 to 0.60% (0.02 SEM) when using data from session 1, 2 and 3. Du et al. [16] compared two adaptation strategies to cope with EMG disturbance for lower limb pattern recognition for prosthetic control. One strategy was based on entropy to determine the confidence the classifier had in its prediction. The other strategy was Learning From Testing data (LIFT), where multiple binary classifiers were trained to determine suited data to update the classifier. They found that by updating the classifier over time, the error rate decreased by 6.5–12% compared with a non-adaptive version. No significant differences were found between the two update methods. Liu et al. [29] extended this analysis by comparing entropy based sample selection with LIFT and transductive support vector machines. They concluded that entropy based sample selection worked best in two able-bodied subjects and one transfemoral amputee and evaluated their method in real-time on another transfemoral amputee. The real-time results showed that entropy based adaptation leads to a better performing prosthesis. Spanias et al. [17] developed a backward predictor called Gait Pattern Estimator, to relabel steps and to update the forward predictor in lower limb pattern recognition used in a transfemoral prosthesis. The authors compared updates based on mechanical sensor information and the Gait Pattern Esitmator, which uses data from the previous stride to update. They found that using the Gait Pattern Estimator, the error rate did not differ significantly from updating using the ground truth labels (resp. 4.5% versus 3% error). The authors applied this update technique in an online setting with transfemoral amputees as well [18]. Using the adaptive system they reduced the error rate from 4.4% to 4.0% for the forward predictions, using a backward predictor with an error rate of 1.6%. Woodward et al. [20] used the backward predictor as well, using a feed forward Neural Network for the forward and backward predictors. They measured four transfemoral amputees over four sessions. After each session they added the session to the training set using labeled data. They compared this approach against using an user independent model and adaptively updating this model using backward prediction. They showed that the backward prediction approach performed similarly. It can be seen that adaptation strategies are beneficial for the lower limb.

The aforementioned studies have shown that updating classifiers after training is crucial for long-term usage of pattern recognition systems. Currently, studies looking into long-term usage in the lower limb are limited and also limited in the number of days and number of subjects. It is unclear which adaptation strategy would be most suited for multi-day pattern recognition in the lower limb. Two sample selection techniques are of interest based on literature. The first strategy is based on entropy, using entropy to determine whether a sample is suited to retrain on. The second strategy is a backward prediction strategy to label new samples, such as the Gait Pattern Estimator by Spanias et al. [17]. In this work, we introduce a third strategy, which is a combination of the two strategies. In the work of Spanias et al. [17] the backward predictor itself was not updated and could possibly suffer from concept drift as well. Therefore, we propose to use entropy for sample selection for the backward predictor over time. The expectation is that by updating the backward predictor, the accuracy of the forward predictor could be improved.

The goal of this study was to investigate concept drift in pattern recognition in the lower limb used on multiple days and compare three adaptation strategies to counter this concept drift. Using a multi-day measurement set-up, a clear picture could be created of how algorithms behave in long-term use and which adaptation strategy would be suited to use in multi-day pattern recognition in the lower extremity.

## 2. Material & Methods

### 2.1. Experimental Data

Ten able-bodied subjects (sex: 4m, 6f; age: 24 ± 2 years; weight: 71 ± 9 kg; height: 174 ± 6 cm) participated in this study. The protocol was reviewed and approved by Medical research Ethics Committees United (MEC-U) Nieuwegein, the Netherlands. The participants provided their written informed consent to participate in this study.

Bipolar EMG was recorded from eight muscles on both legs: rectus femoris, vastus lateralis, biceps femoris, semitendinosus, gluteus maximus, adductor magnus, gastrocnemius medialis and tibialis anterior. All EMG electrodes were placed according to SENIAM guidelines [30]. EMG was recorded using Cometa Wave electrodes at a sampling frequency of 2000 Hz. EMG was filtered with a zero-lag second order butterworth highpass filter with a cut-off frequency of 20 Hz. Lower body kinematics were collected using an MVN Link suit (Xsens, Enschede, The Netherlands), using eight inertial measurement units (IMUs) to reconstruct lower body kinematics at 240 Hz. IMUs were placed on the sternum, pelvis, both thighs, shanks and feet. All data were time synchronized and resampled to 1000 Hz. In this study we used acceleration and angular velocity of the thigh, shank and foot, knee angle, ankle angle and EMG from eight muscles all of the right leg.

**Figure 1:**
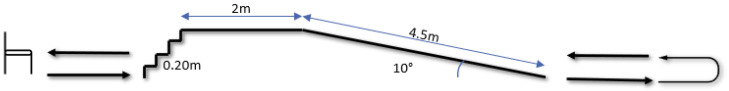
Schematic outline of the measurement set-up. The subject started on the stool, stood up, walked to the stairs, passed over the stairs/ramp combination, walked, turned and walked back to the ramp, passed over the stairs/ramp combination, walked and sat down again. the ramp angle was 10 degrees, the stair step height was 20 cm, stair step length was 22 cm.

Data were collected at the Roessingh Research & Development, Enschede, the Netherlands. Each subject was measured four times: three measurements were conducted on three subsequent days on day 1, 2 and 3 and the last measurement was four days later on day 7. The subjects were measured during the same time slot on each day. Before each measurement the maximal voluntary contraction of each muscle was measured to normalize EMG. The normalization of EMG was done according to the guidelines of Rutherford et al. [31]. Each muscle was contracted for 3 seconds with 3 seconds interval, repeated 3 times. Hereafter, the subjects were asked to perform a circuit of activities, including level-ground walking, stair ascent/descent, ramp ascent/descent and sit-stand motions, see Figure 1. The subject stood up from a stool, walked, ascended the stairs, walked, descended the ramp, walked, turned around, walked back to the ramp, ascended the ramp, walked, descended the stairs, walked and sat down again. This circuit was performed 40 times. Total measurement time including subject preparation, sensor placement and calibration was around three hours per measurement day. After each measurement, an observer labelled the data with the performed activity manually based on kinematics. These labels were assigned by looking at the knee joint angles, ankle angles and a stick figure representation based on the virtual markers. For instance, stair ascent can clearly be recognized by looking at the knee angle in the sagittal plane. If a subject was transitioning from one activity to the next, the timestamp was labelled as the next activity, similar how other online datasets were labelled such as the ENABL3S dataset by Hu et al. [32]. These manual labels were considered the ground truth labels. The data set contained 40 trials per subject per day, resulting in 160 trials per subject, 1600 trials in total. For each subject, there were on average 956 ± 70 samples per day. Each trial was approximately 90 seconds.

### 2.2. Feature Extraction and Classification

We used a mode-specific Neural Network classifier, comparable as described by Woodward et al. [19,20]. The neural network contained one hidden layer with 20 nodes as proposed by Woodward et al. [19] and relu activation. The neural networks were trained using Adam optimizer with a learning rate of 0.001. Neural networks were trained for 100 epochs. If the error rate of the validation set did not increase in three epochs, the training was stopped early. The validation set consisted of 10% randomly chosen samples from the training set. Mode-specific means that for each mode (i.e., a gait related activity) a separate classifier was trained. A window of 300 ms was extracted from each sensor channel prior to each gait event. From each window various features were extracted and these feature vectors were used in the classifier. The features were mean, standard deviation, maximal value, minimal value, start value and end value for the mechanical sensor data and the mean absolute value, zero crossings, slope sign changes, waveform length and coefficients of a fourth order autoregressive model for EMG.

### 2.3. Adaptation Strategies

Based on literature we identified two possible sample selection strategies for pattern recognition adaptation and proposed one novel strategy:

- sample selection based on confidence using entropy [14,16,29];
- sample labelling based on backward prediction [17,18,20];
- combination of backward prediction and updating using entropy.

These sampling strategies will be compared using no adaptation strategy (baseline) and against retraining using the ground truth labels (perfect retraining). Perfect retraining could be considered the most optimal situation, but is not feasible in a real-life setting due to the lack of ground truth labels.

#### 2.3.1. Entropy-Based Sample Selection

**Figure 2:**
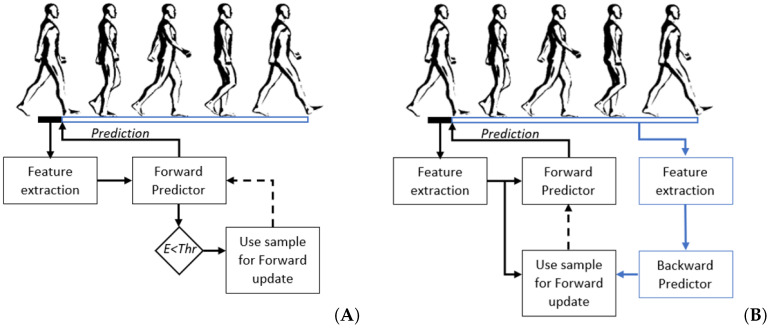
Overview of the adaptation pipelines. (**A**) Entropy based adaptation. First features from a window prior to initial contact were extracted. Hereafter, the forward predictor predicted the upcoming activity. Based on the probability of the forward predictor the entropy (E) is estimated. If the entropy lies below the threshold (Thr) the sample is stored to be used for updating the forward predictor. (**B**) Backward predictor adaptation. The forward prediction works the same as before. After a stride, features were extracted from the previous stride and used by the backward predictor to predict the previous activity. The prediction of the backward predictor was then stored together with the features from the forward prediction to be used for updating the forward predictor.

Entropy is a measure of classifier confidence in its prediction. The lower the entropy, the more confident a prediction is. Entropy (*E*) is defined as [29]:

(1) $$E=-\sum_{k=1}^{N}p_{k}\ln\left(p_{k}\right)$$

where $p_{k}$ is the posterior probability of class *k* out of *N* classes. In this case we used the posterior probability estimated the neural network. The entropy threshold for sample selection was proposed by Du et al. [16] and used by Liu et al. [29]. They found that an entropy value of 0.6 for 5 classes work most optimal. However, in our case, the number of classes differed per mode, we set the entropy threshold to 0.6/ln(5)×ln(N). Overview of the entropy based adaptation pipeline is shown in Figure 2A.

#### 2.3.2. Backward Predictor

Backward predictions were made using a backward predictor as proposed by Spanias et al. [17] and used by Woodward et al. [20]. The backward predictor classified each step after it occurred, as opposed to classifying the motor intent before each step (forward prediction). The backward predictions can then be used as labels for previous samples which can be added to the training set of the forward predictor. The backward predictor existed of a Neural Network classifier, as described before. Features were extracted from mechanical sensor information collected over the previous stride, with a maximal window size of 1500 ms. The extracted features were the mean, standard deviation, maximal value, minimal value, start value and end value. Overview of the backward predictor based adaptation pipeline is shown in Figure 2B.

#### 2.3.3. Backward Predictor with Entropy Adaptation

**Figure 3:**
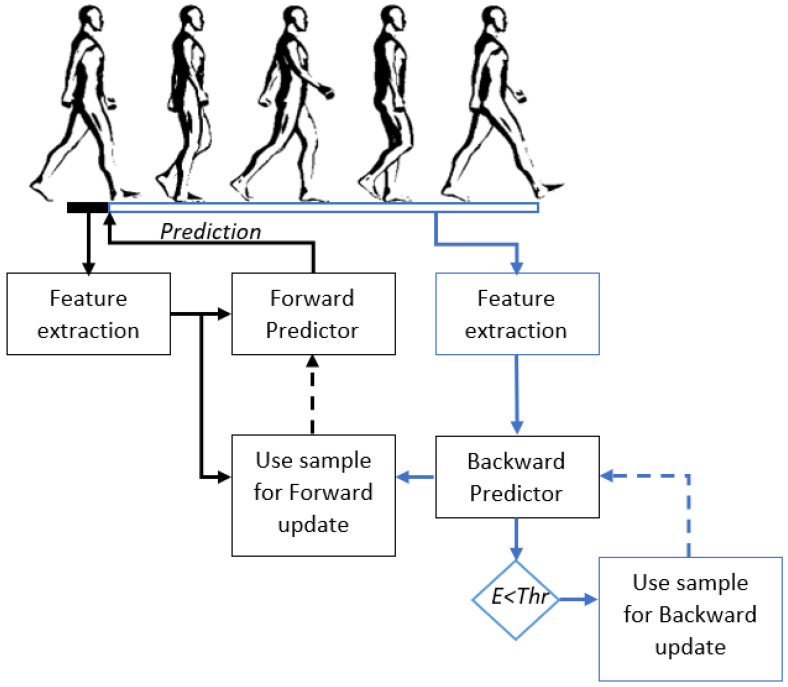
Overview of the backward predictor with entropy adaptation pipeline. First features were extracted from the window before initial contact. The forward predictor used this features to predict the upcoming activity. After a stride the backward predictor predicted the activity of the previous stride. The prediction by the backward predictor was then saved together with the features used by the forward prediction. This sample could then be used for updating the forward predictor. Entropy of the backward prediction was determined using the posterior probability of the backward prediction. If the entropy (E) was below the threshold (Thr) the sample was used for updating the backward predictor.

The next step is to combine the previous described methods. Backward prediction would suffer from concept drift as well, as no adaptation over time takes place. Therefore, we propose to adapt the backward predictor, based on entropy. The posterior probability of the backward prediction is used to calculate entropy. The training set of the backward predictor was extended if a sample had a lower entropy value than the threshold. This threshold was the same as described in the section entropy. After each two trials the backward predictor was updated. Overview of the backward predictor with entropy adaptation pipeline is shown in Figure 3.

### 2.4. Evaluation

In total, there were approximately 160 trials per subject, 40 trials per day. Each predictor was trained on 50% of the data of day 1. The remaining data were used as test set. When a sample was selected or labelled by one of the adaptation strategies, the sample was added to the training set. The predictor was retrained after each two trials. Hereafter, the updated predictor was used to predict the samples of the next trial in the test set, until all trials were used for testing and updating. This means that no sample used for updating was used for training before testing.

To visualize the possible concept drift, we used a principal component analysis (PCA) on the extracted features. This PCA was fitted on data of day 1, retaining two components for easier visualization. Data from the other days was projected on the same principal components.

The evaluation metric used in this work was error rate. Error rate is defined as the number of wrongly classified samples divided by the total number of samples. We used a repeated measures ANOVA with Bonferroni correction to compare the error rates per adaptation strategy and to compare between strategies per day. Normality of the residuals was visually confirmed. Normality of the underlying error distribution was assumed as the number of samples per subject was large.

## 3. Results

### 3.1. Forward Prediction

The results of the repeated-measures ANOVA showed that there was a statistical significant effect of adaptation strategy (F(4,36) = 17.5, *p* < 1 × 10${}^{-4}$, $\eta_{p}^{2}$ = 0.66), of day (F(3,27) = 11.8, *p* < 1 × 10${}^{-4}$, $\eta_{p}^{2}$ = 0.56) and of the interaction between adaptation strategy and day (F(12,108) = 5.3, *p* < 1 × 10${}^{-4}$, $\eta_{p}^{2}$ = 0.37).

**Figure 4:**
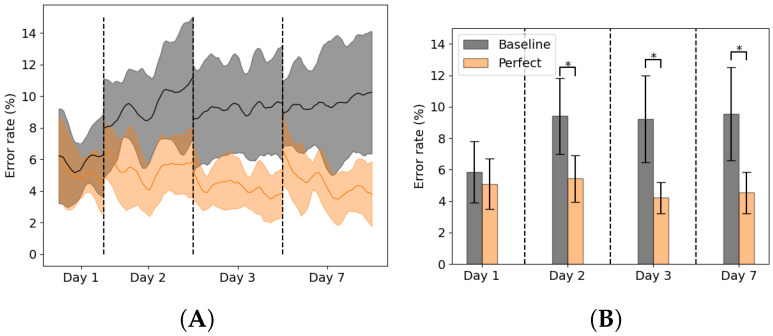
Average forward prediction error rates over all subjects per day for the baseline error rate (gray) and retraining using perfect labels (orange). (**A**) The average error rate over the measurement day. (**B**) The average error rate per day. Asterisk indicates significant difference.

Baseline and retraining using ground truth labels results are shown in Figure 4A,B. When no adaptation was used, the error rates were 5.9 ± 2.0% on day 1, 9.4 ± 2.4% on day 2, 9.2 ± 2.8% on day 3 and 9.6 ± 3.0% on day 7. The error rate increased significantly from day 1 to 2 (*p* = 0.001), but no significant differences were found between the error day 2, 3 and 7. When adaptation was used with ground truth labels the error rates were 5.1 ± 1.6% on day 1, 5.4 ± 1.5% on day 2, 4.2 ± 1.0% on day 3 and 4.5 ± 1.3% on day 7. No significant differences were found between error rates per day.

**Figure 5:**
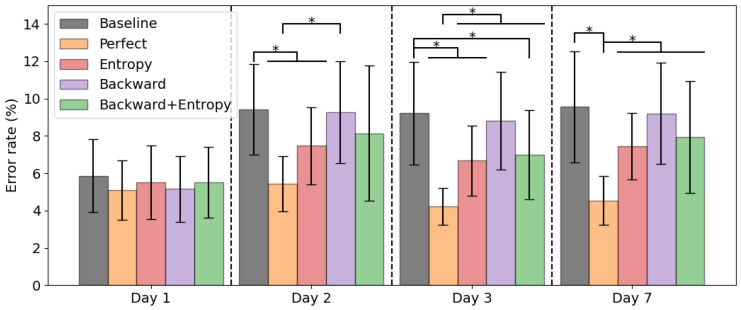
Average forward prediction error rates with standard deviation of baseline and adaptation strategies per day. Asterisk indicates significant difference.

Results of the adaptation strategies are shown in Figure 5. Using entropy based adaptation the error rates were 5.5 ± 2.0% on day 1, 7.5 ± 2.1% on day 2, 6.7 ± 1.9% on day 3 and 7.4 ± 1.8% on day 7. No significant differences were found between error rates per day. Using the backward predictor the error rates were 5.2 ± 1.8% on day 1, 9.3 ± 2.7% on day 2, 8.8 ± 2.6% on day 3 and 9.2 ± 2.7% on day 7. The error significantly increased from day 1 to 2 (*p* = 0.022), but no significant difference was found between day 2, 3 and 7. Using the proposed backward predictor with entropy adaptation the error rates were 5.5 ± 1.9% on day 1, 8.1 ± 3.6% on day 2, 7.0 ± 2.4% on day 3 and 7.9 ± 3.0% on day 7. No significant differences were found between error rates per day. The perfect labelling strategy significantly reduced the error rate compared to the baseline approach on day 2, 3 and 7 (resp. *p* = 0.004, *p* = 0.001, *p* = 0.001), as well as the backward prediction approach on day 2, 3 and 7 (resp. *p* = 0.012, *p* = 0.006, *p* = 0.002). Perfect labelling also reduced the error rate compared to the entropy based method on day 3 and 7 (resp. *p* = 0.001, *p* = 0.001) and the backward prediction with entropy adaptation method on day 3 and 7 (resp. *p* = 0.014, *p* = 0.028). The entropy based method significantly reduced the error rate compared to baseline on day 2 and 3 (resp. *p* = 0.001, *p* = 0.006).

### 3.2. Forward Prediction per Activity

**Figure 6:**
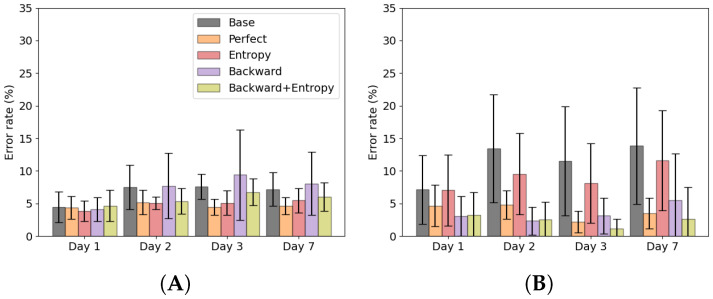
Average forward prediction error rates over all subjects per day per strategy during walking (**A**) and stair ascent (**B**).

**Figure 7:**
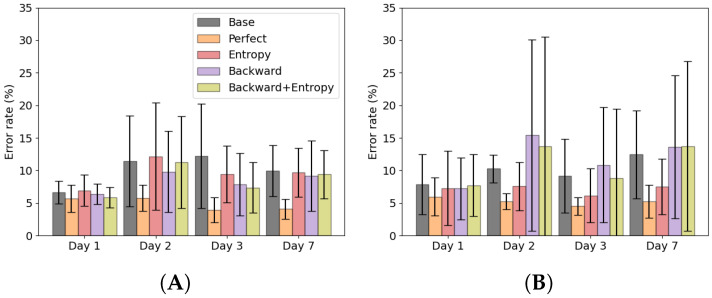
Average forward prediction error rates over all subjects per day per strategy during ramp ascent (**A**) and ramp descent (**B**).

Average error rates of the forward prediction are shown in Figure 6 and Figure 7. When looking at the error rate during walking, Figure 6A, it can be seen that the entropy based adaptation strategy and the backward prediction with entropy adaptation strategy are able to reduce the error compared to baseline. The backward estimation without adaptation showed an increase in standard deviation compared to baseline. For stair ascent, Figure 6B, both backward prediction adaptation strategies show low error rates, comparable to the perfect retraining strategy. Entropy based adaptation shows lower errors than baseline, but performed worse than the other strategies.

In Figure 7A, it can be seen that all adaptation strategies perform comparable to each other and to baseline, which means no improvement is shown. In Figure 7B, it can be seen that the both backward prediction based adaptation strategies show no improvement compared to baseline. Entropy based adaptation shows improvement compared to baseline.

**Figure 8:**
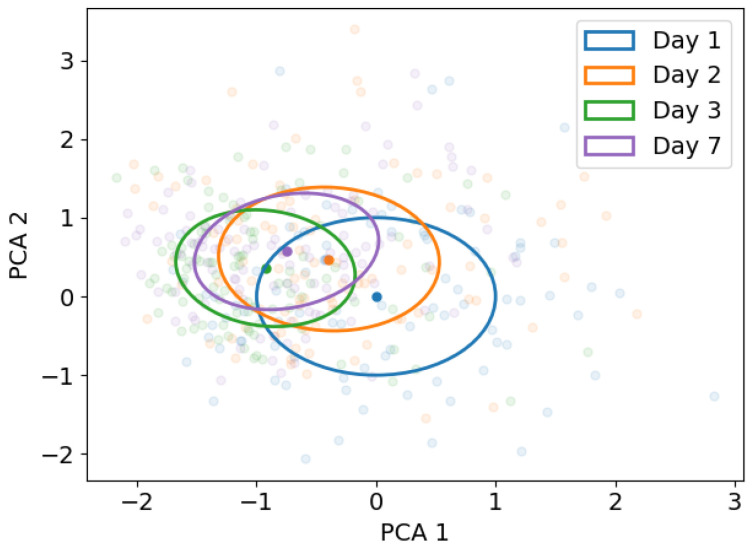
Projection of the extracted features on the first two principal components of day 1 during ramp walking of day 1 (blue), day 2 (orange), day 3 (green) and day 7 (purple) of one subject. Ellipses indicate one standard deviation from the mean, mean values are indicated with the darker dot.

In Figure 8, the projection of the extracted features on the first two principal components of the extracted features of day 1 during ramp walking are shown. The principal component projection forms a visualization of the input data and might give an indication of the variance and changes input data. The variation is largest on day 1, the shift of the mean is largest from day 1 to day 2.

### 3.3. Backward Prediction

The results of the repeated-measures ANOVA showed that there was a statistical significant effect of backward predictor strategy (F(1,9) = 14.6, *p* = 0.004, $\eta_{p}^{2}$ = 0.62), of day (F(3,27) = 8.7, *p* < 1 × 10${}^{-4}$, $\eta_{p}^{2}$ = 0.56) and of the interaction between backward predictor strategy and day (F(3,27) = 2.4, *p* = 0.016, $\eta_{p}^{2}$ = 0.31).

**Figure 9:**
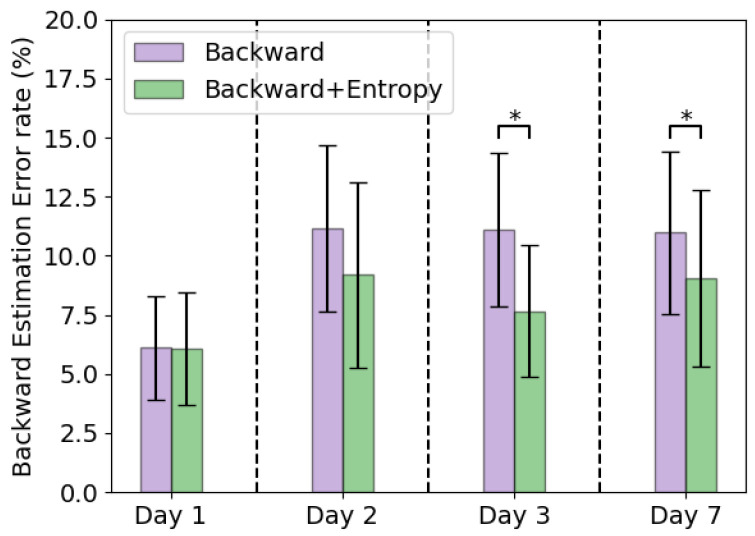
Average error rates with standard deviation of the backward prediction error of the backward predictor and the backward predictor with entropy adaptation. Asterisk indicates significant difference.

The backward predictor error rates are shown in Figure 9. Comparing the backward predictor with the backward predictor with entropy adaptation, it can be seen that the backward predictor using entropy adaptation has a lower backward error rate. Average error rates of the backward predictor were 6.1 ± 2.2% on day 1, 11.2 ± 3.5% on day 2, 11.1 ± 3.2% on day 3 and 11.0 ± 3.4% on day 7. When using the entropy based adaptation, the backward prediction error was reduced on day 2, 3 and 7. The average error rates were 6.1 ± 2.4% on day 1, 9.2 ± 3.9% on day 2, 7.6 ± 2.8% on day 3 and 9.0 ± 3.7% on day 7. The entropy adaptation method significantly reduces the error rate compared to the non-adapted version on day 3 and 7 (resp. *p* = 0.006, *p* = 0.036).

## 4. Discussion

The goal of this study was to address the increase in error rate due to concept drift in pattern recognition in the lower limb, by investigating three adaptation strategies. These adaptation strategies were based on entropy, backward prediction and a combination of these two strategies. The latter was an adaptation strategy we proposed in this study. The baseline error rate increased significantly from day 1 to 2, but plateaus after day 2, showing no significant differences in error rate between day 2, 3 and 7. None of the adaptation strategies was as good as using the ground truth labels. The error rate of the entropy based adaptation strategy and the backward predictor with entropy adaptation strategy showed no significant differences between day 1 and the other days. The entropy based adaptation strategy significantly decreased the error rate on day 2 and 3 compared to baseline.

This study confirms that lower limb pattern recognition suffers from concept drift, as seen by the increase in the baseline error rate and the shift of the mean and covariance of the projections on the principal components with respect to day 1 in Figure 8. Especially the significant increase in error from day 1 to 2 seen in the baseline error rate could harm clinical use. The error significantly increased from 5.9 ± 2.0% on day 1 to 9.4 ± 2.4% on day 2, which is a relative increase of 59%. After day 2 the error seems to plateau and does not differ significantly between day 2, 3 and 7. This might also be explained by the shift in variation seen in the principal components, as the variation seems to reduce and input data became more similar over time, but differed from the input data from day 1.

Using ground truth labels the error rate remained stable around 4–5%. In Figure 4 could be seen that using the ground truth labels also decreased the error rate within one measurement day, especially on day 7. However, the ground truth labels are not available in a real-life setting, which would make this approach not feasible. The alternative is to use entropy based adaptation or a backward predictor with entropy adaptation to counter this concept drift to some degree. Using entropy adaptation the error increased from 5.5 ± 2.0% on day 1 to 7.5 ± 2.1% on day 2, which is a relative increase of 36%. Using the backward predictor with entropy adaptation the error increased from 5.5 ± 1.9% on day 1 to 8.1 ± 3.6% on day 2, which is a relative increase of 47%. Backward prediction suffers from concept drift as well as can be seen in Figure 9. This would mean that such an approach is not feasible for longer periods of time as the backward prediction error increases. This would lead to wrongly updated training set for the forward predictor and ultimately lead to increase of error in the forward predictions. Entropy based adaptation for the backward predictor decreases the effect of concept drift and reduces the backward prediction error rate compared to the non-adapted version. However, compared to entropy-based adaptation, there is no clear benefit on the overall error rate of this approach. Figure 6 and Figure 7 show that the different strategies have different effects on the error rates. The backward prediction strategies both show low error rates on stair ascent. Probably stair ascent is easily recognizable over a stride, for instance in knee angle or large EMG activity, and thus samples can be more reliably relabeled, leading to lower error rates for stair ascent. The opposite might be the case for ramp related activities, where the activities are too similar to walking, which lead to miss labeled samples and higher error rates. The idea that ramp walking is more difficult to recognize is seen in other studies as well, where ramp walking had a relatively higher error compared to stair climbing or where a similar control strategy was used for ramp climbing and level-ground walking [33,34]. The entropy based adaptation showed lower error on walking, stair ascent and ramp descent and comparable results on ramp ascent. Although the relative increase of entropy-based adaptation remains high, it is lower than the relative increase of baseline error of 59%. These activity specific results gives rise to the idea that using different adaptation strategies for different activities might lead to a more a better performing adapting pattern recognition system. For instance, using backward prediction with entropy adaptation during stair climbing and using entropy-based adaptation for other activities. These entropy-based adaptation strategies can be implemented in real-time and might be most suited to be used in further development of lower limb pattern recognition systems as after initial training no manual interventions would be necessary.

The baseline error rate seen in day one, 5.9 ± 2.0%, is comparable with results in literature. Zhang et al. [23] reached an error rate of 15.5 ± 8.0% using an non-optimized neural network and an error rate of 5.1 ± 0.45% using an optimized version in eighty able-bodied individuals performing various gait-related activities. Wang et al. [35] reported an error rate of 2.7–4.8% using a support vector machine for gait mode recognition in eight able-bodied subjects. Zhang and Tao [24] showed error rate of 7.8% using support vector machine and 2.8% using convolutional neural networks in six able-bodied subjects performing various gait-related activities. These studies did not report findings on long-term evaluation of lower limb pattern recognition. Looking at multi-day studies, our results are comparable to that of Du et al. [16] and Liu et al. [29]. Liu et al. [29] showed that the error rate increased from 7.3% to 14.9% when no adaptation strategy was used, which is comparable to this work. Futhermore, the authors showed that the error rate remained below 10% when adaptive strategies were used, comparable to our findings. Zheng et al. [22] showed that by using an automatic labelling strategy based on template matching that the inter-day accuracy could go up from 60% to 88.8%, which emphasizes the need for updating a pattern recognition system when using it over longer periods of time. Spanias et al. [18] found that backward prediction outperformed entropy-based adaptation, which is in contrast with findings in this study. Possible cause for this difference is the accuracy of the backward predictor. The backward predictor of Spanias et al. [18] had an error rate of 1.6%. Our backward predictor had an error rate of 6.1 ± 2.2% on day 1, which increased to 9.2 ± 3.9% on day 2. The large error rate would mean that a forward predictor was incorrectly updated and thus would lead to higher errors. However, the reason for the difference in error rate remains unknown. Possibly, the number of trials used to train the backward predictor were not sufficient and more trials would have been necessary. In a clinical setting this would mean that more training and calibration would be necessary which might not be feasible, although using data from multiple days does reduce the classification error [28]. Another strategy might be to use user independent data [36,37,38,39,40,41] and train the backward predictor on earlier collected data from other users, such as proposed by Woodward et al. [20]. This approach seems to reduce the initial error of the backward predictor and leads to better forward predictor updating. Next to that activity recognition or locomotion mode recognition could play a role to enhance labelling of samples [42,43], although this requires longer windows of data, possibly increasing the computational load. Another approach would be the use of additional sensors such as depth sensing for terrain identification to update labels [44,45]. These changes could lead to a better backward predictor and therefore probably lead to a better forward prediction. In its current state the error rate of the backward predictor is too high to result in reliable labeling for the adaptation of the forward predictor.

One of the limitations of this study is the use of able-bodied subjects instead of patients groups, such as amputees with a controllable prosthesis. However, as we only intended to show the influence of concept drift and the differences between various adaptation strategies, the use of able-bodied subjects is justified. Our results are generated on a larger subject population than described before in literature spanning multiple days, but the amount of subjects is still limited. The sample size of ten subjects is small, although the $n_{p}^{2}$ > 0.14 which indicated a large effect size for model, day and the interaction effect. This suggests that the sample size was adequate for our research. However, additional data using patient groups as well, could be helpful to provide a clearer result between the different adaptation strategies. However, these results give insight into the error rate in lower limb pattern recognition over multiple days and show the necessity of implementing adaptation strategies. Next to this, the MVC that was used for EMG normalization cannot easily be performed by amputees or patients. However, for clinical use, a submaximal contraction, as for instance during walking, could be used to estimate maximum contraction [46]. The major advantage of this submaximal contraction is that it can also be estimated during daily use, thus circumvents the need for additional daily calibration routines. However, we did not investigate this approach in a multi-day setting and future research should verify whether submaximal contractions could be used for EMG normalization in multi-day lower limb pattern recognition. The next step would be to compare the most promising strategies over multiple days in a patient group, such as amputees, to confirm clinical benefit. At the moment it is unclear whether these results would be good enough for multi-day pattern recognition to be used in clinical settings, as the increase in error rate is still substantial over days. Future work could focus on robustness of feature sets by selecting more optimal feature sets as shown by Wang et al. [35] or focus on more deep learning algorithms which have the opportunity to model variances over time [47]. Selecting optimal features might lead to more robust performance [35,37,48], by optimizing feature selection for a specific problem. However, in this work, we have only focused on the adaptation strategies and chosen to use a feature set which was commonly described in literature. Next to that future work should focus on home monitoring of users during a multi-day study, to see whether the entropy based adaptation strategy would still be most optimal to use in this scenario.

## 5. Conclusions

In this work, we investigated three adaptation strategies to address concept drift in multi-day pattern recognition. We evaluated the three approaches on a data set containing ten able-bodied subjects, consisting of 40 trials per day evaluated over 4 measurement days, of which three were consecutive and the final day was 7 days after the first measurement day. It can be concluded that the baseline error rate increased significantly from day 1 to 2, but remained stable over the other measurement days. Entropy based adaptation showed the smallest increase in error rate and can be considered to be the most feasible adaptation strategy for multi-day pattern recognition overall, although the backward predictor using entropy adaptation showed promising results for predicting stair ascent and descent, giving the rise to the idea to combine multiple error compensation strategies. The results of this study indicate the necessity of multi-day measurements to evaluate lower limb pattern recognition to be used in multi-day control.

## Data Availability

The data presented in this study are openly available in MyPredict 3. 4TU.ResearchData Collection. 10.4121/c.6094686.
